# Pain’s impact on eating patterns and inflammation: A case-control study

**DOI:** 10.1097/MD.0000000000039492

**Published:** 2024-09-06

**Authors:** Kübra Şahin, Metin Saip Sürücüoğlu, Müge Arslan

**Affiliations:** a Nutrition and Dietetics, Faculty of Health Sciences, Uskudar University, Saray, Ahmet Tevfik İleri St. Nu:5 Umraniye, Istanbul, Turkey; b Nutrition and Dietetics, Faculty of Health Sciences, International Cyprus University, Nicosia, Cyprus; c Nutrition and Dietetics, Faculty of Health Sciences, Uskudar University, Saray, Ahmet Tevfik İleri St. Nu:5 Umraniye, Istanbul, Turkey.

**Keywords:** nutrition disorders, overnutrition, pain

## Abstract

Pain is a widespread and troubling clinical and social problem with important effects on society and individuals. The purpose is to assess the relationship between pain and eating behavior, macro-micronutrient intake, and dietary inflammation index. The study was carried with a total of 80 patients, consisting of 40 patients diagnosed with pain and 40 patients not diagnosed with pain, who applied to a private hospital in Istanbul as outpatients with a questionnaire face-to-face consisting of questions about sociodemographic characteristics, anthropometric measurements, pain-related information, Eating Attitude Test (EAT-19), and 24-hour food consumption record. The statistical analysis of the data was conducted with SPSS v27 package program. People who had pain had higher levels of disrupted eating attitudes than those who did not have pain. The “Bulimia” subfactor mean score of the EAT-19 was higher in those with pain (*P* < .05). No difference was found between the case-control groups regarding the mean dietary inflammation index (DII) score and energy, macro- and micronutrient consumption values (*P* > .05). No difference was detected between the case-control groups with disrupted eating attitudes regarding the median DII score (*P* > .05). The median DII score was significantly higher in individuals with pain and normal eating attitudes than in those without pain and with disrupted eating attitudes (*P* < .05). There is a relationship between pain, eating attitudes, and DII, the median DII score of those who had pain and had normal eating attitudes was higher.

## 1. Introduction

Pain is a widespread and troubling clinical and social problem with important effects on the society and individuals.^[1]^ The International Association for the Study of Pain defines pain as an emotional experience related to possible tissue damage.^[2]^ The World Health Organization estimates that about 10% to 20% of patients applying to primary health care services complained of pain and 22% stated that their pain persisted.^[3]^

By reducing functional capacity and the quality of life and making patients less physically active, pain affects eating attitudes.^[4]^ As a result of the increase in pain, people may consume foods with simple sugar content and high energy value, and eating behaviors can be altered and deteriorated.^[5]^ In a study conducted by Nezamoleslami et al,^[6]^ it was found that patients who had pain had unhealthy eating attitudes when compared to the healthy controls. In a study by Emir et al,^[7]^ it was demonstrated that patients who had pain had higher eating attitude scores in comparison to healthy controls. In the study by Zick et al^[8]^ regarding the relationship between pain and macro-micronutrient intake, the patients who were diagnosed with pain had higher carbohydrate consumption when compared to the control group. In a study by Philpot et al,^[9]^ it was found that low intake of micronutrients such as B1, B3, B6, B12, vitamin D, and β-carotene in the diet was related to inflammation and pain. Furthermore, in a study conducted by Pagliai et al,^[10]^, insufficient vitamin C consumption was associated with pain.

Nutrition and pain processes interaction may result from the dietary effects on inflammation, which is the widely hypothesized mechanism for pain, and the content of dietary inflammation can impact pain intensity.^[11]^ The source, type, and amount of carbohydrates, proteins, and fats from macronutrients and excess energy intake influence inflammation parameters. Refined carbohydrate consumption, glycemic index, foods high in fat and trans fat, and cholesterol intake lead to increased release of proinflammatory cytokines such as C-reactive protein, tumor necrosis factor-alpha, interleukin (IL)-6, and IL-1β. Unlike saturated and transfatty acids, monounsaturated fatty acid and polyunsaturated fatty acid exhibit protective activity against inflammation.^[12]^ Previous studies have indicated that there is a relationship between decreased consumption of foods and nutrients with anti-inflammatory properties and increased inflammation and pain.^[13,14]^

The aim of this study is to investigate the effect of pain on eating patterns and inflammation. The present study is significant because it reveals the relationship between pain, which is associated with the progression of many illnesses and has an influence on the individuals’ life quality and comfort, with nutrition and inflammation, which has a great impact on the processes of disease formation and treatment, and it reveals the inadequate and unbalanced nutritional status caused by the nutritional change that develops along with the pain. Moreover, this study is a reference for further studies to be carried out in this field because of the lack of literature on studies in which pain-related changes in eating attitudes, food intake, and dietary inflammatory index are investigated in combination.

## 2. Materials and methods

The study population consisted of a total of 446 adult outpatients who were admitted to the Physical Therapy and Rehabilitation Department of Erdem Hospital between November 2021 and May 2022 as outpatients and were diagnosed with “pain” by a doctor.

Using the number of patients diagnosed with pain in the previous year of the hospital, the power of the test was taken as 1-β-0.80 with type 1 error level α = 0.05 and type 2 error level β = 0.05, and the statistical power analysis was calculated as 34 people using GPower 3.1.0 statistical programme. In this context, 40 people who met the study conditions were determined as the case group.

In total, the study was conducted with 80 patients, 40 of whom were diagnosed with pain by a doctor, and 40 of whom were not diagnosed with pain using a simple random selection method.

This volunteer-based study was launched following the Ethics Committee Approval dated November 26, 2021 and numbered 58797649-050 from Istanbul Kent University Research and Publication Ethics Committee. The purpose and scope of the study were explained to the participants and their “Voluntary Participation Consent” was obtained. A questionnaire including questions related to the participants’ sociodemographic features, anthropometric measurements, pain-related information, eating attitude test, and 24-hour food intake record was conducted face-to-face.

Among the anthropometric measurements, height, body weight, and BMI were obtained. The body weight was measured using a calibrated electronic scale, with an empty stomach, dressed in thin clothing, and without shoes. The height was measured with a stadiometer at the Frankfort level without shoes, with the feet side by side. BMI was calculated by divided body weight by square meters of height, and classified according to the “World Health Organization’s (WHO)” BMI classification.

In order to calculate the participants’ macro-micronutrient intake, 24-hour retrospective food intake records were obtained. The mean consumption of daily energy and nutrients (energy, carbohydrate, protein, fat, vitamin, mineral) of individuals were analyzed by using the full version of the “Computer Assisted Nutrition Program, Nutrition Information System 9” program.^[15]^ In order to calculate the dietary inflammation index (DII) of participants, a 24-hour retrospective food consumption record was taken from the participants and the DII score was calculated based on these data. The index was based on human, animal, and cell culture studies examining the impact of various nutrients including nutrients, nutrient components, and flavonoids on inflammation. This is a literature-based index developed by examining the effect of dietary components on serum proinflammatory and anti-inflammatory markers IL-1β, IL-4, IL-6, IL-10, tumor necrosis factor-alpha, and C-reactive protein.

It was developed in 2014 by Shivappa et al in order to assess the anti/proinflammatory impact of the consumed diet. By accessing data from various national nutrition surveys in different parts of the world, Shivappa et al obtained the average daily consumption and standard deviation values of 45 nutrient parameters. To calculate the DII, the daily consumption of each nutrient by individuals was subtracted from the global average consumption of each nutrient and then the z score was calculated by dividing by the value of the standard deviation for that nutrient. Once the z score was converted into a percentage score, it was multiplied by the “nutrient-specific full inflammatory effect score” to find the DII score based on the consumption of that nutrient. The same procedure was followed for 45 foods, and the total DII score of the individual was determined. The DII scores were divided into quartile groups in equal numbers and named as first quartile (Q1), second quartile (Q2), third quartile (Q3), and fourth quartile (Q4). The first quartile (Q1) corresponds to an anti-inflammatory diet. As the quartiles increase, inflammatory load of the diet rises and the fourth quartile (Q4) corresponds to a proinflammatory diet. Positive score is evaluated as increasing inflammation, while negative score is evaluated as reducing inflammation and zero point is considered as having no effect on inflammation.^[16]^

To measure the eating attitudes and behaviors of the participants, EAT-19 was used. Assessment factors of EAT-19 were defined as “Obesity Anxiety (items 1, 8, 9, 10, 11, 16, 18), Eating Obsession (items 3, 4, 14, 15), Dieting (items 5, 6, 12, 13, 17), Bulimia (items 2, 7, 19).” In scoring the test, “always is 3 points, “usually is 2 points, “frequently is 1 point and “sometimes, rarely and never is 0 points. Points are obtained by totaling the answers given to the questions. The cutoff value is 9.5. Those with a score of 9.5 or above are regarded as “unhealthy and tendency to abnormal eating behaviors whereas those with a score below 9.5 are regarded as “tendency to normal eating behaviors.”^[17]^

### 2.1. The statistical analyses used in the study

For the categorical variables (demographic features), the descriptive statistics were presented as frequency and percentage. The suitability of numerical variables for the normal distribution was controlled with the “Shapiro–Wilk Test.” Mean ± SD (X ®±SD) values were given for normally distributed data and median (min-max) values were given for non-normally distributed data. “Independent sample *t* test” was used for the comparison of 2 independent groups with normal distribution and “Mann–Whitney *U* test” was used for the comparison of 2 independent groups without normal distribution. The statistical significance level was considered as “*P* < .05, *P* < .01, *P* < .001” in all the calculations and evaluations in the study and hypotheses were formed bidirectionally. The statistical analysis of the data was carried out in “SPSS v27 (IBM Inc, Chicago, IL, USA)” package program.

## 3. Rwesults

The mean age of the individuals in the study case group was 41.80 ± 11.79 years, the mean BMI was 26.15 ± 4.22 kg/m^2^ and 60.0% had a BMI of ≥ 25 kg/m^2^, 75.0% of them were married, 40.0% of them were university graduates, 27.5% of them were insured workers, 97.5% of them missed the evening meal, and 52.5% of them had a disrupted eating attitude (Table 1).

**Table 1:** The demographic, health, habit, and nutritional characteristics of individuals by case-control groups and gender.

	Case group (n = 40)						Control group (n = 40)					
	Male (n = 18)		Female (n = 22)		Total (n = 40)		Male (n = 20)		Female (n = 20)		Total (n = 40)	
	n	%	n	%	n	%	n	%	n	%	n	%
Age (yrs) ($\bar{X}\pm SS$)	40.67 ± 11.59		42.73 ± 12.14		41.80 ± 11.79		38.75 ± 11.81		40.00 ± 10.85		39.38 ± 11.21	
BMI (kg/m^2^) ($\bar{X}\pm SS$)	25.95 ± 4.62		26.32 ± 3.97		26.15 ± 4.22		26.32 ± 3.80		24.15 ± 3.22		25.24 ± 3.65	
BMI group												
<25 kg/m^2^*	9	50.0	7	31.8	16	40.0	8	40.0	11	55.0	19	47.5
≥25 kg/m^2^†	9	50.0	15	68.2	24	60.0	12	60.0	9	45.0	21	52.5
Marital status												
Married	15	83.3	15	68.2	30	75.0	12	60.0	10	50.0	22	55.0
Single	3	16.7	7	31.8	10	25.0	8	40.0	10	50.0	18	45.0
Education status												
Illiterate	-	-	1	4.5	1	2.5	-	-	-	-	-	-
Literate	-	-	-	-	-	-	-	-	-	-	-	-
Primary school	3	16.7	4	18.2	7	17.5	1	5.0	-	-	1	2.5
Middle school	1	5.6	4	18.2	5	12.5	-	-	-	-	-	-
High School	3	16.7	5	22.7	8	20.0	3	15.0	4	20.0	7	17.5
University	9	50.0	7	31.8	16	40.0	14	70.0	14	70.0	28	70.0
Master’s degree/PhD	2	11.1	1	4.5	3	7.5	2	10.0	2	10.0	4	10.0
Occupation												
Government official	-	-	-	-	-	-	-	-	-	-	-	-
Insured worker	8	44.4	3	13.6	11	27.5	8	40.0	12	60.0	20	50.0
Self-employment	3	16.7	1	4.5	4	10.0	4	20.0	2	10.0	6	15.0
Retired	-	-	1	4.5	1	2.5	3	15.0	3	15.0	6	15.0
Housewife	-	-	9	40.9	9	22.5	-	-	1	5.0	1	2.5
Teacher	1	5.6	2	9.1	3	7.5	-	-	1	5.0	1	2.5
Unemployed	-	-	2	9.1	2	5.0	-	-	-	-	-	-
Engineer	3	16.7	1	4.5	4	10.0	4	20.0	-	-	4	10.0
Student	2	11.1	1	4.5	3	7.5	1	5.0	-	-	1	2.5
Doctor	1	5.6	-	-	1	2.5	-	-	-	-	-	-
Academician	-	-	2	9.1	2	5.0	-	-	1	5.0	1	2.5
Meal skipping situation‡												
Morning	17	94.4	19	86.4	36	90.0	17	85.0	17	85.0	34	85.0
Noon	11	61.1	14	63.6	25	62.5	15	75.0	14	70.0	29	72.5
Evening	18	100.0	21	95.5	39	97.5	20	100.0	20	100.0	40	100.0
Mid-morning	2	11.1	3	13.6	5	12.5	6	30.0	6	30.0	12	30.0
Afternoon	7	38.9	13	59.1	20	50.0	10	50.0	13	65.0	23	57.5
Night	6	33.3	6	27.3	12	30.0	8	40.0	6	30.0	14	35.0
Eating behavior disorder situation												
Disrupted eating attitudes	6	33.3	13	59.1	19	47.5	7	35.0	9	45.0	16	40.0
Normal eating behavior	12	66.7	9	40.9	21	52.5	13	65.0	11	55.0	24	60.0

BMI = body mass index.

* Since the sample size was small, this group was statistically combined and evaluated within the normal BMI category.

† Since the sample size was small, this group was statistically combined and evaluated in the overweight BMI category.

‡ More than 1 answer was given.

In the control group, individuals had a mean age of 39.38 ± 11.21 years, a mean BMI of 25.24 ± 3.65 kg/m^2^, 52.5% had a BMI of ≥ 25 kg/m^2^, 55.0% were married, 70.0% were university graduates, 50.0% were insured workers, 100.0% missed the evening meal, and 60.0% had a disrupted eating attitude (Table 1).

In the “Bulimia” subfactor scores of the EAT-19, the median (0 [0–2]) of the individuals in the case group was found to be statistically higher than the median (0 [0–1]) of those in the control group (U = 678.5; *P* < .05) (Table 2).

**Table 2:** The comparison of EAT-19 subfactor, total scores, and DII scores of individuals with the case-control groups.

	Case group	Control group	t-U	*P*
	$\bar{X}\pm SD$ Median (min–max)	$\bar{X}\pm SD$ Median (min–max)		
EAT subfactors				
Anxiety about fatness	6 (0–15)	3 (0–21)	U = 620	0.081
Obsession with eating	0 (0–7)	0 (0–8)	U = 742.5	0.488
Diet	1.5 (0–11)	2.5 (0–12)	U = 766.5	0.742
Bulimia	0 (0–2)	0 (0–1)	U = 678.5	0.025*
EAT–19_total	9 (0–30)	6.5 (0–36)	U = 695	0.311
DII	2.9 (−8.6 to 7.9)	1.4 (−8.2 to 7.9)	U = 714	0.408
BMI	26.15 ± 4.22	25.24 ± 3.65	t = 1.037	0.303

BMI = body mass index, EAT = Eating Attitude Test, DII = dietary inflammatory index, t = independent sample *T* test, U = Mann–Whitney *U* test.

* *P* < .05.

There was no statistically significant difference (*P* > .05) between the DII scores and BMI values of the individuals who participated in the study compared to the case-control groups (Table 2).

No statistically significant difference (*P* > .05) was observed between the energy, macro and micro values from the 24-hour food consumption record of the individuals who participated in the study compared to the case-control groups (Table 3).

**Table 3:** The comparison of energy, macro and micro values from 24-hour food consumption records of individuals with the case-control groups.

	Case group	Control group	t-U	*P*
	$\bar{X}\pm SD$ Median (min–max)	$\bar{X}\pm SD$ Median (min–max)		
Energy (kcal)	1301.6 (756–3332.5)	1263.9 (386.1–3174.1)	U = 797	0.977
Water (g)	894.8 (261–1899.1)	865.4 (204.4–3373.3)	U = 790	0.923
CHO (g)	126.1 (33.7–393.7)	111.5 (24–374.5)	U = 690	0.290
CHO (%)	40.28 ± 12.17	35.42 ± 12.76	t = 1.740	0.086
Protein (g)	57.1 (20.5–132.9)	58.4 (20.7–173.9)	U = 716	0.419
Protein (%)	16 (7–25)	17 (11–47)	U = 646.5	0.138
Fat (g)	63 (27.7–113.5)	69.3 (22.4–155.9)	U = 709	0.381
Fat (%)	42.90 ± 10.93	45.58 ± 10.94	t = −1.094	0.277
Saturated fat (g)	23.26 ± 8.19	23.55 ± 10.77	t = −0.135	0.893
Monounsaturated fat (g)	22.4 (8.2–49.8)	24.8 (8.3–68)	U = 674	0.225
Polyunsaturated fat (g)	16.98 ± 9.15	18.04 ± 9.58	t = −0.505	0.615
Omega-3 (g)	1.87 ± 2.03	1.61 ± 0.97	t = 0.750	0.456
Omega-6 (g)	13.3 (1.9–34.4)	16 (2.5–40.9)	U = 735	0.532
Cholesterol (mg)	268.78 ± 162.73	283.98 ± 153.46	t = −0.430	0.669
Fiber (g)	18.52 ± 7.58	16.90 ± 7.92	t = 0.936	0.352
Vitamin A (µg)	819.1 (244.1–4415)	838.7 (93–2492.5)	U = 743	0.583
Vitamin D (µg)	1.5 (0–38)	1.5 (0–37.6)	U = 701	0.340
Vitamin E (mg)	16.59 ± 8.06	18.03 ± 9.25	t = −0.744	0.459
Vitamin B_1_ Thiamine (mg)	0.6 (0.3–1.9)	0.6 (0.2–1.3)	U = 772	0.788
Vitamin B_2_ riboflavin (mg)	1 (0.4–2.1)	1 (0.3–2.7)	U = 783.5	0.874
Niacin (mg)	10.2 (3.3–30.6)	10 (4.2–39.8)	U = 712	0.397
Vitamin B_6_ Pyrid (mg)	1.1 (0.4–2.1)	1.2 (0.4–2.6)	U = 721	0.447
Carotene (mg)	1.7 (0.3–9.8)	1.8 (0.3–8.6)	U = 786.5	0.897
Total folate (µg)	204.7 (87.9–492.3)	210.1 (60.1–382.7)	U = 739	0.557
Vitamin B_12_ (µg)	3.3 (0.3–10.8)	3.1 (0–10.5)	U = 773	0.795
Vitamin C (mg)	55.9 (2.1–187.2)	65.6 (7.4–202.1)	U = 759	0.693
Sodium (mg)	3063.1 (987.7–8299.5)	3196.3 (1163.5–9178.6)	U = 713	0.403
Potassium (mg)	1634.7 (688.9–3240.9)	1702.7 (742.6–4846.2)	U = 759	0.693
Calcium (mg)	488.3 (202.6–1735.6)	452.5 (111.3–1286.5)	U = 777	0.825
Magnesium (mg)	204.7 (86.7–640.2)	213.3 (78–576.1)	U = 743	0.583
Phosphorus (mg)	842 (401.7–2012.1)	830.1 (256.8–1918.6)	U = 781	0.855
Iron (mg)	9.8 (4.4–22)	10.1 (4.4–22.7)	U = 680	0.248
Zinc (mg)	7.7 (4–16.9)	8.8 (4–21.1)	U = 659.5	0.176

t = independent sample *T* test; U = Mann–Whitney *U* test.

It was found that the median of individuals with a BMI of ≥ 25 kg/m^2^ in the case group (6.5 [0–15]) was statistically higher than the median of individuals with a BMI of ≥ 25 kg/m^2^ in the control group (3 [0–12]) regarding the “Fat Anxiety” subfactor scores of the EAT-19 (Table 4).

**Table 4:** The comparison of EAT-19 subfactor and total scores and DII scores of participants based on case-control groups and gender, BMI group, and eating behavior disorder status.

	BMI group	Case group	Control group	t-U	p
		$\bar{X}\pm SD$ Median (min–max)	$\bar{X}\pm SD$ Median (min–max)		
EAT subfactors					
Anxiety about fatness	<25 kg/m^2^	4.5 (0–9)	5 (0–21)	U = 146	0.841
	≥25 kg/m^2^	6.5 (0–15)	3 (0–12)	U = 143.5	0.013*
Obsession with eating	<25 kg/m^2^	0 (0–3)	1 (0–8)	U = 97.5	0.037*
	≥25 kg/m^2^	0 (0–7)	0 (0–7)	U = 212	0.211
Diet	<25 kg/m^2^	1.5 (0–11)	3 (0–12)	U = 128	0.417
	≥25 kg/m^2^	1.5 (0–9)	2 (0–9)	U = 235	0.692
Bulimia	<25 kg/m^2^	0 (0–2)	0 (0–0)	U = 114	0.023*
	≥25 kg/m^2^	0 (0–2)	0 (0–1)	U = 232	0.356
EAT-19 total	<25 kg/m^2^	9 (0–18)	9 (0–36)	U = 136	0.596
	≥25 kg/m^2^	9 (0–30)	6 (0–16)	U = 171.5	0.066
DII	<25 kg/m^2^	1.9 (−8.6 to 7.1)	2.8 (−8.2 to 7.3)	U = 150	0.947
	≥25 kg/m^2^	3.3 (−8.1 to 7.9)	−0.7 (−7.2 to 7.9)	U = 212	0.363
	Eating behavior disorder status				
DII	Disrupted eating attitudes	−0.27 ± 5.88	1.49 ± 5.01	t=−0.944	0.352
	Normal eating attitude	4.7 (−6.1 to 7.2)	−0.9 (−8.2 to 7.6)	U = 162	0.041*

EAT = Eating Attitude Test, DII = dietary inflammatory index, t = independent sample *T* test, U = Mann–Whitney *U* test.

* *P *< .05.

It was found that the median (1 [0–8]) of individuals with a BMI of < 25 kg/m^2^ in the control group was statistically higher than the median (0 [0–3]) of individuals with a BMI of < 25 kg/m^2^ in the case group in the “Obsession with Eating” subfactor scores of EAT-19, and it was found that the median of the “bulimia” subfactor scores of individuals with a BMI of < 25 kg/m^2^ in the case group (0 [0–2]) was statistically higher than the median of individuals with a BMI of < 25 kg/m^2^ in the control group (0 [0–0]).

No statistically significant difference (*P* > .05) was detected among the DII scores of the participants according to the case-control groups (Table 4).

Among the DII scores, it was found that the median of the individuals with normal eating attitudes in the case group (4.7 [−6.1 to 7.2]) was statistically higher than the median of the individuals with impaired eating attitudes in the control group (−0.9 [−8.2 to 7.6]) (Table 4).

There was no statistically significant difference (*P* > .05) between the DII scores of the individuals with disrupted eating attitudes who participated in the study according to the case-control groups (Table 4).

## 4. Discussion

In the present study, the mean and median BMI of people who had pain were found to be higher than those who did not have pain. In the study conducted by Basem et al^[18]^ regarding the effect of obesity level measured by BMI on pain and pain severity, the mean BMI of patients of both sexes who were diagnosed with pain was 27 kg/m^2^, and in the study carried out by Jakoniuk et al,^[19]^the mean BMI of the patients was 28.1 kg/m^2^ in the case group and 26.2 kg/m^2^ in the control group. In a similar way, in studies by Zick et al^[8]^ and Ferreria et al,^[20]^ the mean BMI of individuals with pain was also found to be higher than that of individuals without pain. This can be attributed to the fact that high body weight may cause extreme load on the spine and joints and increase the risk of pain.

In the present study, individuals who had pain mostly skipped the morning meal, whereas those who did not have pain mostly skipped the evening meal. Yilmaz ^[21]^ reported that the most common meal skipped in both groups was the morning meal. The pain may cause sleep disorders and accordingly, people with pain may have difficulty waking up in the morning. In this case, this may cause people with pain to skip the morning meal.

In this study, individuals who had pain had more disrupted eating attitudes compared to individuals who did not have pain. Likewise, similar studies have demonstrated that patients diagnosed with pain have higher EAT scores and unhealthy eating attitudes when compared to healthy controls.^[6,7]^ Differently, Bulut et al^[22]^ showed that the uncontrolled eating scores were higher in the control group and patients with pain did not have any negative eating behaviors. The pain may affect eating habits in a negative way.^[23]^ It is emphasized that this type of eating habits and eating attitudes influence the risk of pain in patients, increasing obesity, exacerbating pain symptoms and decreasing functional capacity.^[24,25]^ This situation may lead to a disrupted eating attitude with skipping meals due to pain, refusing to eat or overconsumption of food to deal with pain.

The higher median of bulimia subfactor in individuals with pain may be explained by the fact that they may change their eating attitudes depending on the severity of pain, skip meals and show bulimic behavior at the next meal due to excessive food intake due to hunger.

In the present study, the mean DII score of the individuals who had pain was higher than the individuals who did not have pain. Similarly, studies have reported that patients with a diagnosis of pain had higher DII scores than the control group.^[11,26–28]^ Individuals may have difficulty in their movements due to the type and severity of pain, and depending on this, they may tend to use prepackaged products rather than preparing meals. In this situation, the DII is high with the intake of processed foods, which are high in energy and saturated fat content and contain basic sugars.

In the present study, individuals with pain had higher energy intake than individuals without pain. According to similar study, patients diagnosed with pain in the case group had higher energy intake than the control group.^[6]^ Differently, studies have indicated that individuals with pain have lower energy intake than individuals without pain.^[8,29,30]^ According to the study conducted by Gio et al^[24]^, energy restriction shows that it has positive effects on pain symptoms by inhibiting inflammation. This situation can be explained with the fact that individuals who have pain tend to eat to deal with pain based on the severity and frequency of pain and their energy intake increases due to increased food consumption.

In the present study, the mean consumption values of carbohydrate (g) and carbohydrate (%) from water and macronutrients were higher in individuals with pain than in individuals without pain. In similar study, the patients diagnosed with pain had higher carbohydrate consumption than the control group.^[8]^ Differently, in the study conducted by Nezamoleslami et al^[6]^, those with pain diagnosis had less carbohydrate consumption compared to the control group. This situation can be explained in the sense that because pain causes water loss through sweating due to stress, individuals may tend to consume more water to compensate for water losses and may tend to turn to carbohydrate-containing foods to deal with pain.

In the present study, the mean consumption values of protein g, protein %, fat g, fat %, saturated fat g, monounsaturated fat g, polyunsaturated fat g, omega (n)-6 and cholesterol from macronutrients were found to be higher in individuals who did not have pain than in individuals who had pain. Likewise, in a study conducted by Yilmaz^[21]^, fat (g, %) consumption in the patient group with pain diagnosis was lower than in the control group. In other studies, the mean dietary protein (g) and fat (g) consumption of patients with pain diagnosis was higher than the control group.^[6,8]^ Due to the anxiety of pain that may be experienced by individuals without pain, they could tend to consume foods with high protein and fat content such as meat, milk and eggs to strengthen joint and muscle health.

In the present study, the mean consumption of n-3 g in individuals who had pain was higher than that in individuals who did not have pain. Likewise, in a study conducted by Yilmaz,^[21]^ the mean n-3 consumption value was found to be higher in the patient group with pain diagnosis compared to the control group. Carballo-Casla et al,^[31]^ consumption of 1.5 serving of oily fish per week reduced pain scores by 0.68 point. This situation can be explained by the high consumption of n-3 fatty acids, which has anti-inflammatory characteristics, in individuals with pain to relieve their pain.

In the present study, the mean consumption of fiber g of individuals who had pain was found to be higher than that of individuals who did not have pain. Likewise, fiber intake was higher in the patient group diagnosed with pain compared to the control group in the study conducted by Yilmaz.^[21]^ Differently, in the study conducted by Zick et al,^[8]^ those with pain diagnosis had less fiber consumption compared to the control group. This situation can be explained by the fact that people with pain may have consumed more fiber because fiber has anti-inflammatory properties, reducing inflammation and relieving pain. There is a need for more case-control studies examining nutritional habits in studies conducted on patients diagnosed with pain.

In the present study, the mean values of vitamin A, vitamin D, vitamin E, vitamin C, vitamin B1, vitamin B2, vitamin B2, vitamin B6, folate, and carotene consumption from micronutrients were found to be lower in individuals who had pain in comparison to individuals who did not have pain. In a study by Philpot et al,^[9]^ it was observed that low dietary consumption of micronutrients like vitamin B1, vitamin B3, vitamin B6, vitamin B12, vitamin D, and β-carotene was related to inflammation and pain. In a study by Yilmaz et al^[21]^, it was found that the mean dietary vitamin E intake of individuals with a diagnosis of pain was lower compared to individuals without pain. In a different way, vitamin C intake mean intake was found to be higher in individuals with pain. In a meta-analysis study, it was found that 500 mg/day of vitamin C supplementation for 50 days decreased pain syndrome.^[32]^ In a meta-analysis performed by Joustra et al,^[33]^ it was stated that vitamin E levels were lower in patients suffering from pain than in healthy controls. In studies, it was established that inadequate levels of vitamin C were correlated with musculoskeletal pain.^[9,10]^ In the study by Yilmaz,^[21]^ the level of vitamin D intake in the patient group with pain diagnosis was found to be higher than in the control group. In the study by Helde et al,^[34]^, it was observed that vitamin D was found to be effective in the severity and sensitivity of pain. Studies on vitamin D supplementation have demonstrated a significantly greater decrease in pain scores in patients with chronic pain using vitamin D supplementation when compared to placebo, suggesting that vitamin D supplementation may play a role in the management of chronic pain.^[35,36]^ In patients with pain, it is known that B-group vitamins have anti-inflammatory efficacy and decrease pain scores in musculoskeletal pain.^[37]^ Since vitamins have antioxidant properties, they can reduce inflammation in the body. This can be explained by the fact that vitamin-deficient diets will cause pain by increasing inflammation.

In the present study, the mean values of consumption of sodium, potassium, magnesium, iron, and zinc from the micronutrients were found to be lower in individuals who had pain than in individuals who did not have pain. In a study by Philpot et al,^[9]^ a low dietary intake of micronutrients including magnesium and zinc was associated with inflammation and pain. In the central nervous system, zinc is present in excess, regulates pain, and is implicated in the pain process via N-methyl d-aspartate receptors.^[37]^ It has been documented that adults who have pain consume less magnesium than those who do not have pain.^[30]^ Furthermore, previous studies have demonstrated that supplementation of the diet with magnesium would be related to lower levels of inflammation and relieve pain.^[26,38]^ By blocking the ion channel in the N-methyl d-aspartate receptor, which is physiologically involved in neuronal impulse transmission, it prevents extracellular calcium from penetrating into the cell and thereby provides secondary neuronal changes. By this mechanism, pain neurons’ activity can be decreased by preventing central sensitization.^[30,39]^ Antioxidant features of minerals and their effect on the functions of the nervous system cause positive results on pain.^[9,40]^ This could be attributed to the fact that mineral-deficient diets increase inflammation, causing damage to tissues and leading to pain.

Among the eating attitude subfactors, the median score of anxiety about fatness was significantly higher in individuals who had pain and had a BMI ≥ 25 kg/m^2^ than in individuals who did not have pain and had a BMI ≥ 25 kg/m^2^. This situation can be explained by the increase in the body weight of individuals who have pain depending on the change of pain in eating attitudes, and they may experience anxiety fat. Individuals with a BMI < 25 kg/m^2^ who did not have pain had a higher mean score of obsessive eating, a subfactor of eating attitudes, than individuals with a BMI < 25 kg/m^2^ who had pain. This situation can be explained by their more obsessive eating behavior because of the anxiety about the possible effects of pain. The mean score of bulimia, which is one of the eating attitude subfactors, was higher in individuals who had pain and BMI < 25 kg/m^2^ than in individuals who had no pain and BMI < 25 kg/m^2^. This can be explained by the fact that individuals who have pain have a vomiting tendency because of excessive food intake in the next meal depending on meal skipping. Individuals with pain and a normal eating attitude had higher DII scores than those without pain and with a normal eating attitude. This can be explained by the fact that due to the movement difficulties associated with the current pain, they tend to turn to practical packaged foods that are processed, contain high saturated and transfat content, and contain simple sugars, causing an increase in DII scores. There is a need for studies examining the relationship between BMI and eating attitudes in patients with and without a diagnosis of pain.

This study is a reference for further studies to be conducted in this field due to the lack of literature on studies in which pain-related eating attitudes, food intake, and dietary inflammatory index were investigated together and compared with people without pain diagnosis. Weaknesses of the study include the difficulty in reaching a larger sample size because of the difficulty in accessing patients with pain diagnosis due to the fact that cases were taken from private hospitals.

## 5. Conclusions

Eating habits are a changeable lifestyle factor related to pain. Strategies for healthy eating have a major role in pain management. Treatment of patients in pain should involve diet therapy as well as basic medical treatment.

## Acknowledgments

The authors thank all participants involved in the study.

## Author contributions

**Conceptualization:** Kübra Şahin, Metin Saip Sürücüoglu, Müge Arslan.

**Data curation:** Kübra Şahin, Müge Arslan.

**Formal analysis:** KKübra Şahin, Metin Saip Sürücüoglu.

**Funding acquisition:** Kübra Şahin.

**Investigation:** Kübra Şahin.

**Methodology:** Kübra Şahin.

**Project administration:** Kübra Şahin.

**Resources:** Kübra Şahin.

**Software:** Kübra Şahin.

**Visualization:** Kübra Şahin.

**Writing—original draft:** Kübra Şahin, Metin Saip Sürücüoglu, Müge Arslan.

**Supervision:** Metin Saip Sürücüoglu, Müge Arslan.

**Validation:** Metin Saip Sürücüoglu, Müge Arslan.

**Writing—review & editing:** Metin Saip Sürücüoglu, Müge Arslan.
